# Sequential Extraction and Characterization of Essential Oil, Flavonoids, and Pectin from Industrial Orange Waste

**DOI:** 10.1021/acsomega.4c00112

**Published:** 2024-03-16

**Authors:** Dilara Nur Dikmetas, Dilara Devecioglu, Funda Karbancioglu-Guler, Derya Kahveci

**Affiliations:** Faculty of Chemical and Metallurgical Engineering, Department of Food Engineering, Istanbul Technical University, Maslak 34469, Istanbul, Turkey

## Abstract

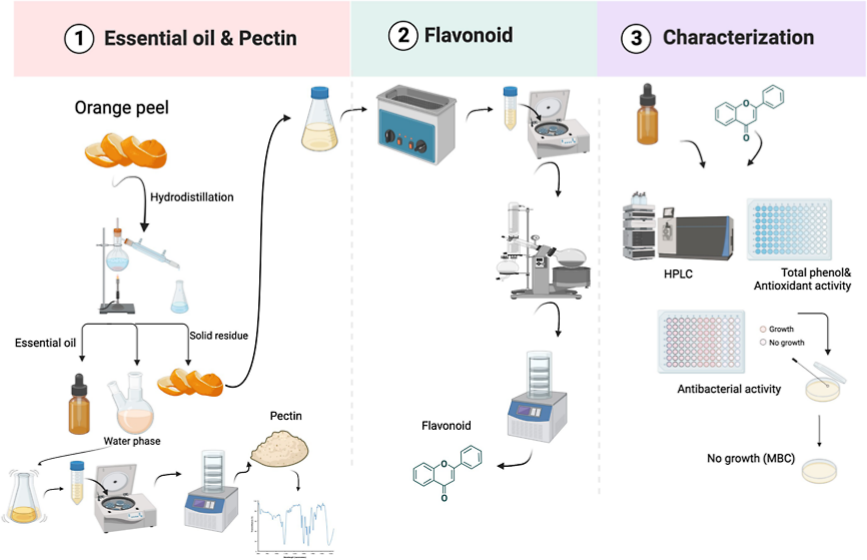

Orange is one of the primary fruits processed into juice and other products worldwide, leading to a vast amount of waste accumulation. Such waste has been considered as an attractive candidate for upcycling to obtain bioactive components remaining. The present study investigated the extraction of essential oil (EO), flavonoids, and pectin from industrial orange waste with a holistic approach. To maximize EO yield and d-limonene concentration, hydrodistillation (HD) conditions were selected to be 5.5 mL water/g solid for 180 min. Remaining solids were further used for flavonoid extraction where conventional solvent, sequential ultrasound + solvent, and ultrasound-assisted extraction (UE) were applied. UE applied for 50 min with 120 mL solvent/g solid yielded the highest total phenolic (TPCs) and total flavonoid contents (TFCs), antioxidant capacity, and hesperidin and neohesperidin concentrations. In terms of TPC, TFC, antioxidant capacity, and antibacterial activity, both EO and flavonoid fractions demonstrated moderate to high bioactivity. At the final step, ethanol precipitation was applied to obtain the pectin that was solubilized in hot water during HD and it was characterized by Fourier transform infrared, degree of esterification, and galacturonic acid content. Practical application: to ensure utilization in the food, pharmaceutical, and cosmetic industries, this study presents a combined method to obtain several value-added compounds from industrial orange waste. Bioactive EO and flavonoids obtained could have applications in functional food, supplements, or cosmetic formulations, whereas extracted pectin can be used in many formulated foods and drugs.

## 1 Introduction

Orange (*Citrus sinensis*) represents the major citrus fruit produced globally, accounting to 83.2 million tonnes in 2021, 175 million of which was produced in Turkey.^1^ Several processed food products, such as juice, jams, marmalade, etc., are produced from approximately 1/3 of oranges produced, leading to a vast amount of waste. It has been reported that orange waste accumulates at around half of the total weight of oranges,^2^ mostly composed of flavedo, albedo, pulp, and seeds. Solid waste management has been said to be “the most important concern in citrus-processing industries”.^3^ Orange waste is high in fermentable carbohydrates and moisture and has antibacterial properties, both of which are making the traditional landfilling treatment problematic.^4^ Additionally, the 2018/850 directive of the EU states that solid waste that has not been treated for energy or resource recovery cannot be diverted to landfill. Therefore, alternative approaches to handling orange waste need to be considered. A typical of those approaches involves upcycling of orange waste to be used as animal feed; however, it lacks an adequate amount of protein and is low in pH and has been suggested to require modification to improve its suitability for feedstock.^5^ On the other hand, value-added ingredients for food, pharmaceutical, and cosmetic applications, such as essential oil (EO), polyphenols, and pectin, have been produced from orange peel (OP) obtained from orange processing for decades, well before “upcycling” was defined.

Orange EO is mainly composed of monoterpenes that are produced as secondary metabolites, about 90% of which is d-limonene.^6^ Several bioactive functions of orange EO, including antibacterial,^7^ antifungal,^8^ antioxidant,^7,9^ antidiabetic,^10^ and anticarcinogenic effects,^11^ have been reported. It is stated that limonene and *p*-cymene, which are contained in the EOs obtained from OP, can be economically feasible with their high financial value.^12^ Orange-derived polyphenols, flavonoids in particular, are also of great interest due to several bioactive functions. Main components of flavonoid fraction obtained from OP are naringin, hesperidin, and rutin.^13^ This fraction, especially hesperidin, has been emphasized for having antimicrobial, antioxidant, anticarcinogenic, and antidiabetic activities, and it is the major flavonone in citrus fruits.^4,14−16^ Pectin is one of the most abundant components remaining in fruit and vegetable waste and has been traditionally produced from citrus peels and pomace to be used as a thickener and emulsifier in formulated foods, as well as in the pharmaceutical industry for the production of many drugs.^17^

The orange juice industry is a dynamic sector in the food industry that generates a lot of waste, as more than half of processed fruit is known to be discarded. Based on the reduce, reuse, recycle, recover, and restore (5R) principle of sustainable development, the circular economy concept describes the use of waste from one industry as a raw material to another, replacing the traditional linear model of the economy (make-use-throw) with a much more effective circular model.^18^ Furthermore, the concept of bioeconomy, which is explained by the utilization of renewable biological resources into economically valuable products and bioenergy, has emerged.^3^ Therefore, the current study suggests that orange juice industry leftovers could make excellent feedstock for the generation of bioactive components, fulfilling the goals and objectives of the circular economy. Additionally, the orange juice industry also is an ideal feedstock for the production of energy.^19^ From this point of view, several approaches exist for the valorization of citrus waste, and OP in particular, to extract the above-mentioned components for food, pharmaceutical, and cosmetic applications.^3,20,21^ Most of these attempts concentrate on obtaining a single fraction (EO, phenolic compounds or pectin) from such waste. However, the processing of industrial waste with a holistic approach that allows obtaining as much material as possible is important in terms of waste reduction and sustainable and economical production.^22−24^ However, some components included in the wastes limit their utilization. Since the limonene-rich EO contained in orange waste decreases its usage potential, EO must be extracted. Consequently, while a high added value component is obtained, it is possible to obtain more than one fraction by utilizing the waste separated after the extraction process.^25^ Existing literature on extraction of more than one bioactive component from citrus waste includes fermentation,^26^ microwave-assisted solvent-free^27^ or water extraction,^28^ steam-distillation followed by acidic extraction,^29,30^ and Ohmic heating-assisted hydrodistillation (HD).^31^

The present work focused on procedures that could be scaled up, such as conventional HD, ultrasound-assisted solvent extraction (UAE), and ethanol precipitation, to obtain EO, flavonoids, and pectin from OP. UAE is an environmentally friendly extraction technique that uses less energy and solvent, requires less time and money for extraction, and yields a greater product recovery rate than traditional techniques.^32^

Tamminen et al.^33^ highlighted that scaling up the sonochemical process to continuous or large-scale operations is a critical barrier for industrializing the UAE technology. However, there have been few published reports of industrial or experimental processing plants in the literature.^33−36^

The primary objective of the current study is to sequentially extract EO, flavonoid, and pectin from orange waste. However, various components from OP, as opposed to the literature, primarily focused on the extraction of single fraction from orange waste, such as pectin,^1−4^ flavonoids,^38^ and d-limonene.^39^ EO and flavonoid fractions were especially focused on due to their biological activity. Therefore, extraction routes for these two components were optimized to maximize the yield of d-limonene and selected flavonoids, and the bioactive functions (antimicrobial and antioxidant activities, total phenolic compounds, and flavonoid concentrations) were investigated. Finally, the structural characterization of extracted pectin was reported. Pectins are extensively utilized in the food and medical industries. The are primarily utilized as a gelling agent, thickening, emulsifier, and stabilizer in the food sector to enhance the quality of food items. Pectin can also serve as a material for developing edible and coated films due to its excellent biodegradability, biocompatibility, and diverse physicochemical features, apart from its role in food production.^40^

Hence, the main goal was to discover a practical application for the excess orange waste that is unsuitable for consumption and is generated in large quantities during the process of manufacturing orange juice. This waste would be utilized as a raw material for the production of EO, flavonoid, and pectin for different purposes including the food industry, cosmetic, and biomaterial preparation. Instead of the previous efforts in upcycling of such waste that focused on production of a single component, the present work enables the extraction of several components from industrial orange waste, thereby leading to an improved cost efficiency of several steps that must be implemented.

## 2 Materials and Methods

### 2.1 Materials

#### 2.1.1 Plant Materials

Orange (*C. sinensis*) peel (OP) waste originated from Aydin (Aegean region) in 2022 was obtained from industrial fruit juice producers, Dimes Gıda Sanayi ve Ticaret A. Ş. and AEP Anadolu Etap Penkon Gıda ve Tarım Ürünleri Sanayi ve Tic. A. Ş. (Türkiye). OP was stored at −40 °C, and before the experiment, it was coarsely ground by a blender. Compositional analysis revealed that OP was composed of 75.4% moisture, 21.4% carbohydrates, 1.4% protein, 1% lipids, and 0.7% ash. OP was stored at −20 °C until used.

#### 2.1.2 Chemicals

High-performance liquid chromatography (HPLC)-grade acetonitrile, formic acid, methanol, and Folin-Ciocalteu reagent were purchased from Merck (Darmstadt, Germany). HPLC-grade flavonoid standards, sodium carbonate, 1,1-diphenyl-2-picrylhydrazyl (DPPH), Trolox (6-hydroxy-2, 5, 7, 8-tetramethylchroman-2-carboxylic acid), and all of the chemicals and growth media used in the antimicrobial analysis were purchased from Sigma-Aldrich (St. Louis, MO, USA). All other chemicals used were of analytical grade.

### 2.2 Extraction and Characterization of EO

Seventy-five grams of OP were homogenized with distilled water and subjected to HD in a 1 L round-bottom flask connected to a Clevenger apparatus at 100 °C. The crude EO and a few condensation products were collected after extraction. Na_2_SO_4_ was used to dry the EO, totally removing any lingering condensation byproducts, after which both the EO and the solid residue of HD (OP-HD) were kept at −18 °C until further experiments.^41^

To achieve the highest EO yield and d-limonene concentration, the experimental conditions were optimized using a 3^2^ factorial design. Water-to-solid ratio (4, 5, and 7.5, v/w) and HD time (120, 180, and 240 min) were chosen as the factors.

The yield of EO has also been calculated by the following formula^42^
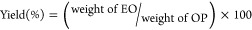


d-limonene concentration was measured by the method of Park et al.,^43^ with slight modifications. HPLC (Agilent, 1100) equipped with a DAD detector and a C18 column (250 × 4.6 mm, 5 μm; Supelco, USA) was used. The column was held at 25 °C. Chromatographic analyses were performed using a 20 μL manual sample injector. The flow rate was 1 mL/min, and the wavelength of the DAD detector was 200 nm (d-limonene chromatogram is available at Figure S1). The mobile phase was composed of 95% methanol. Standard d-limonene at known concentrations was used to determine the compound yield in EO.

A factorial analysis of variance (ANOVA) was conducted to assume the main and interaction effects of the chosen factors on the dependent variables. According to this analysis, an HD duration of 180 min and a water/solid ratio of 5, which were statistically more effective, were selected for further experiments.

### 2.3 Extraction of Flavonoids

#### 2.3.1 Comparison of Flavonoid Extraction Methods

Three different extraction methods, namely, conventional solvent extraction (SE), sequential ultrasound + solvent extraction (USE), and ultrasound-assisted extraction (UE), have been used to maximize the flavonoid concentration, total phenolic compound content, and antioxidant activity. Moreover, the flavonoid profile had also been taken into account. 70% ethanol was used in all approaches since it was reported to be the appropriate solvent to obtain the highest total phenolic and flavonoid content and antioxidant activity.^44−46^

##### 2.3.1.1 Conventional SE

Two grams of lyophilized OP-HD were extracted with 40 mL of 70% ethanol. A mechanical stirrer was used to shake the mixture at 200 rpm in the dark for 30 min at room temperature. After that, flavonoids were obtained by centrifugation at 4 °C and 8000*g* for 10 min, and the supernatant was collected. The same procedure was repeated for a second time by using the remaining pellet. The supernatants were pooled and concentrated in a rotary vacuum evaporator at 40 °C.

##### 2.3.1.2 Sequential USE

Two grams of lyophilized OP-HD in 40 mL of 70% ethanol were ultrasonicated in an ultrasonic bath (VWR USC900TH ultrasonic cleaner, VWR Int. Radnor, PA, USA) for 30 min and subsequently centrifuged at 4 °C and 8000*g* for 10 min, and the supernatant was collected. The remaining pellet was mixed with 40 mL of 70% ethanol and shaken by a mechanical stirrer at 200 rpm for 30 min at room temperature. After that, flavonoids were obtained by centrifugation and concentrated as above.

##### 2.3.1.3 Ultrasound-Assisted Extraction

Two grams of lyophilized OP-HD were extracted with 40 mL of 70% ethanol in an ultrasonic bath for 30 min and subsequently centrifuged at 4 °C and 8000*g* for 10 min. The same procedure was repeated for a second time using the remaining pellet. After that, flavonoids were obtained by centrifuging and concentrated as mentioned above.

#### 2.3.2 Optimization of Flavonoid Extraction Conditions

Depending on the results from Section 2.3.1, the UE was chosen for further experiments. In order to maximize both total phenolic and total flavonoid contents (TPCs and TFCs, respectively) as well as antioxidant activity, the experimental conditions were optimized using a 3^2^ factorial design. Solvent-to-solid ratio (40, 80, and 120, v/w) and total extraction time (50, 60, and 70 min) were chosen as the factors. Table 3 shows the various combinations of these factors as well as the results for the dependent variables.

#### 2.3.3 Quantification of Flavonoids

The contents of *p*-anisidine, rutin, hesperidin, neohesperidin, naringenin, and hesperetin were quantified with modifications of the method by Kim and Lim.^47^ Flavonoids were identified by the same HPLC instrument that is described in Section 2.2 at 270 nm. The mobile phases consisted of 0.05% formic acid in water (A) and 0.05% formic acid in acetonitrile (B). The solvent flow rate was 0.5 mL/min, the injection volume was 10 μL, and the column temperature was set to 30 °C using gradients for of B: 0 min 15%, 8 min 25%, 15 min 25%, and 35 min 65%. TPC and TFC procedures are given below.

### 2.4 Extraction and Characterization of Pectin

Pectin was extracted from the residual aqueous phase of HD by the combined and modified methods by Oliveira et al.^48^ and Saberian et al.^49^ The sample (pH: 1.5) was treated with 95% ethanol (1:1 v/v) at 4 °C for 1 h, and the resulting mixture was centrifuged at 4000 rpm for 7 min to collect the precipitated pectin. After lyophilization, the extraction yield of pectin was calculated as the percentage of pectin (g) to the initial OP (g).

To determine the galacturonic acid (GalA) content of pectin, the method of Blumenkrantz and Asboe-Hansen^50^ was applied with slight modifications. Pectin (5 mg) was mixed with 2 mL of 72% H_2_SO_4_ and 15 mL of distilled water. After mixing for 1 h at room temperature, 3 mL of concentrated sulfuric acid containing 12.5 mM sodium tetraborate was added to the 0.5 mL sample. The mixture was kept in boiling water for 5 min and cooled immediately in a water-ice bath. Then, 50 μL of 0.15% 3-phenylphenol reagent (weight of reagent to volume of 0.5% NaOH) was added, and the mixture was left for 10 min at room temperature. The absorbance was read at 520 nm, and the GalA content was determined according to the standard curve of GalA solutions at 100–600 nmol/mL.

The degree of esterification (DE) of extracted pectin was determined by the titrimetric method of Hosseini et al.^51^ with slight modification. Lyophilized pectin (75 mg) was dissolved in 100 mL of distilled water while stirring. After complete dissolving, five drops of phenolphthalein reagent were added, and the solution was titrated with 0.1 M NaOH (*V*_1_). Then, 20 mL of 0.5 M HCl was added to the sample and left for 15 min at room temperature. The sample was mixed with a magnetic stirrer until the pink color disappeared. After adding five drops of phenolphthalein reagent, the mixture was titrated with 0.1 M NaOH until a slight pink color was observed (*V*_2_). The DE (%) was calculated as
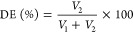


The extracted pectin powders’ spectra of Fourier transform infrared (FT-IR) spectroscopy (Bruker Tensor II, Massachusetts, USA) in the range of 400–4000 cm^–1^ were obtained and compared with the spectra of commercial pectin.

### 2.5 Bioactivity Analysis

EO diluted in ethanol (10 mg/mL) 1% dimethyl sulfoxide (DMSO) and extracts obtained from flavonoid extraction procedures were used for bioactivity analysis.^52^

#### 2.5.1 Determination of TPC

The Folin-Ciocalteu method was used to determine the TPC.^53,54^ Briefly, 0.75 mL of 0.2 N Folin-Ciocalteu reagent (10%) was added to 100 μL of samples. After keeping the samples in the dark for 5 min, 750 μL of saturated Na_2_CO_3_ (6%) solution was added to this mixture. After the samples were vortexed and kept at room temperature in a dark environment for 90 min, absorbance measurements were made at 765 nm in a UV–vis spectrophotometer (BioTek Instruments, Winooski, Vermont, USA). Results were expressed as milligrams of gallic acid equivalent/100 g of the sample and gallic acid equivalent/L of EO.

#### 2.5.2 Determination of TFC

TFC of the EO and flavonoid extracts have been determined by the method by Zhishen et al.^55^ Briefly, 0.25 mL of the sample was mixed with 1.25 mL of distilled water. After that, 75 μL of 5% NaNO_2_ was added. After 6 min, 150 μL of 10% AlCl_3_·6H_2_O was added, and the mixture was stored for 5 min. Finally, 0.5 mL of 1 M NaOH was added and the volume of the mixture was completed to 2.5 mL with distilled water. The mixture was shaken, and the absorbance of the mixture was determined at 510 nm by a UV–vis spectrophotometer. Results were expressed as milligrams of rutin equivalent/100 g extract and milligrams of rutin equivalent/L EO.

#### 2.5.3 Determination of Total Antioxidant Capacity

DPPH and cupric-reducing antioxidant capacity (CUPRAC) methods were used to determine the total antioxidant activities of the EOs and flavonoids. The DPPH method was conducted according to the method by Kumaran and Joel Karunakaran.^56^ 100 μL of the sample was added to 2 mL of a 0.1 mM DPPH solution prepared in 100% methanol. The samples were then kept in a dark room for 30 min. Absorbance of the samples was measured at 517 nm in a UV–vis spectrophotometer. In the CUPRAC analysis, 100 μL of samples was mixed with 1 mL of each of 10 mM CuCl_2_, 7.5 mM neocuprine, NH_4_Ac (pH: 7), and distilled water. After incubation for 30 min at room temperature, the absorbance of the samples was determined at 450 nm by a UV–vis spectrophotometer.^57^ Results were expressed as milligrams of Trolox equivalent/100 g of extract and milligrams of Trolox equivalent/L of EO.

#### 2.5.4 Antimicrobial Activity

The antimicrobial activities of EO and flavonoids were tested against three Gram-positive (*Staphylococcus aureus* ATCC 6538, *Streptococcus pyogenes* ATCC 19615, and methicillin-resistant *S. aureus* ATCC 43300) and two Gram-negative (*Pseudomonas aeruginosa* ATCC 27853 and *Escherichia coli* ATCC 25922) bacteria. All microorganisms were cultured overnight at 37 °C in Mueller-Hinton broth (MHB) and diluted in sterile saline solution (0.85% w/v NaCl) to reach a final concentration of approximately 10^5^ CFU/mL.

The minimum inhibitory concentrations (MICs) and minimum bactericidal concentrations (MBCs) of EO and flavonoids were determined with the broth microdilution technique in a 96-well plate according to modified methods of Chahbi et al.^58^ The stock solutions of EO (512 mg/mL) and flavonoids (128 mg/mL) were prepared in sterilized 10% DMSO including 2% Tween 80 and sterilized 10% DMSO, respectively. 2-Fold diluted EO (4–256 mg/mL) and flavonoid (64–0.0625 mg/mL) concentrations were prepared in MHB. Briefly, 180 μL of the diluted sample and 20 μL of bacterial suspension were transferred to wells. For the samples’ negative control, the mixture of 180 μL of the diluted sample and 20 μL of MHB was used, and wells without the diluted sample were used as the positive control. The MIC value was defined as the lowest sample concentration without turbidity at 600 nm after incubation at 37 °C for 24 h. Then, the mixture in the wells was inoculated on the Mueller-Hinton agar plates, and the concentration with no bacterial growth was defined as MBC. Finally, the results were confirmed by the resazurin assay based on color change principle specified by Foerster et al.^59^

### 2.6 Statistical Analysis

All experiments were reported as mean ± standard deviation of three independent replicates with three parallel measurements. The statistical significance of differences among groups was evaluated by factorial ANOVA, followed by Tukey test using Minitab (Ver. 18.0, USA), and significance was identified with a value of *p* < 0.05.

## 3 Results and Discussion

### 3.1 Extraction and Characterization of EO

EO was extracted from the OP residue in the orange juice industry. The effects of HD time and water-to-solid ratio were investigated on EO yield and d-limonene concentration. The results are shown in Table 1.

**Table 1:** Factorial Design with the Observed Results for the EO Yield and d-Limonene Concentration

HD time (min)	water-to-solid ratio (v/w)	EO yield (%)	d-limonene concentration (%)
120	4	0.29	37.43
120	5.5	0.35	19.19
120	7	0.38	24.68
180	4	0.45	23.84
180	5.5	0.38	20.49
180	7	0.26	21.21
240	4	0.57	13.37
240	5.5	0.44	18.30
240	7	0.48	14.90
120	4	0.32	14.74
120	5.5	0.29	28.83
120	7	0.78	20.53
180	4	0.23	22.27
180	5.5	0.45	20.83
180	7	0.29	23.92
240	4	0.23	16.60
240	5.5	0.73	21.73
240	7	0.47	20.31

**Figure 1:**
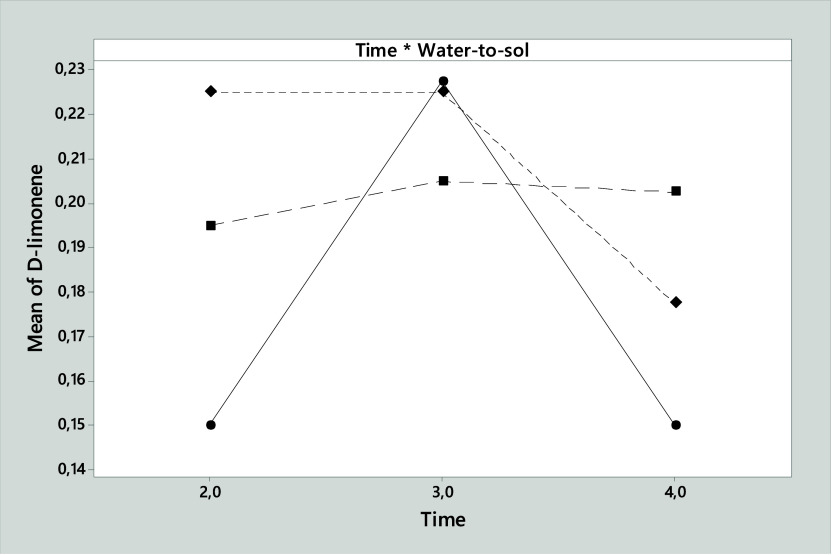
Effect of interaction of water-to-solid ratio (●, 4; ■, 5.5; and ⧫, 7) and time on d-limonene concentration.

According to the findings, water-to-solid ratio and HD time significantly affected d-limonene concentration (*p* < 0.05) but not the EO yield, the latter of which was in contrast to previous studies.^60^ Extraction yield of EO was in the range of 0.29–0.76%; however, no significant differences were observed. Similar to our findings, Mohagheghniapour et al.,^61^ Bourgou et al.,^62^ and Visakh et al.^63^ reported citrus EO yield as 0.20, 0.35, 0.45, and 0.48%, respectively, while the yield of EO has also been reported as 1–3% by several researchers.^60,64−66^ In a recent study by Wei et al.,^67^*Citrus medica* L. var. *arcodactylis* EO yield was reported as 1.29 ± 0.03% with the HD method. These variations can be explained by the fact that the citrus peel waste used in the present study differed.^67^ Another explanation for varying EO yields could be the proper separation of hydrosols, which consists of a small portion of EO that is separated into distilled water during the HD process. This secondary product is formed as a result of hydrogen bonding between polar oil vapors and water during the extended distillation period, thereby leading to loss of some EO yield.^21,68^ The results of the chemical analysis of the EO were consistent with prior studies that d-limonene was the major constituent of orange EO.^60,69^ Orange EO in the present study contained lower d-limonene content than in previous studies^9,60,70,71^ and was accounted as 16.60–37.43%. Similar to our results, Heydari Koochi et al.^10^ reported that sweet orange and bitter OP waste EO contained 19.91 and 18.12% of d-limonene. Soil conditions, management, topography, and climate conditions, including temperature, light, wind, and rainfall, can all have an impact on the quantity and quality of citrus EO. Additionally, the method used to extract the EO impacts the quality.^10^ At 95% confidence level, HD time and water-to-solvent ratio as well as the interaction of these two independent variables (*p* = 0.003) (Figure 1) were found to have statistically significant effects on the d-limonene concentration.

The highest d-limonene concentration was found at 3 h and a water-to-solid ratio of 5.5. Results in Figure 1 show that d-limonene extraction progressively increased up to 3 h, and decreased afterward, at the specified water-to-solid ratio. Fick’s second law of diffusion could be used to explain this as mass transfer of the solute from biomass to solvent phase can only occur until the system reaches equilibrium.^72^ Additionally, because of the characteristics of d-limonene, prolonged extraction times may have caused this unsaturated compound to degrade under the influence of light, heat, and air.^73^

One of the crucial factors influencing HD is the ratio of water-to-solid materials, which is defined as the weight of the material dissolved in a certain volume of the solvent such as water. The effect of the water-to-solid ratio was investigated at 4, 5.5, and 7 g/mL. The low water amount would not be sufficient to diffuse into the material. Additionally, a larger sample amount might enable the extraction of a higher percentage of EO, but it leads to a concentrated sample or poor chemical solubilization because there is only so much solvent available. As a result, the ratio between water and solids should permit the equilibrium concentration to stay below the solute’s saturation concentration.^71,74^ According to Table 1 and Figure 1, the EO yield was unaffected, whereas the concentration of d-limonene declined at higher water-to-solid ratios and was lowest at the lowest level used. Similar to our results, Dao et al.^74^ reported optimum water-to-solid ratio as 3 mL/g when 2, 3, and 4 mL/g were investigated by HD of citrus leaves. The same phenomenon was also explained by several researchers.^31,71,74,75^

In light of results regarding both dependent variables, HD conditions were selected to be 5.5 mL of water/g of solid for 180 min. At these conditions, EO with 0.42 ± 0.05% yield and 20.66 ± 0.24% d-limonene was obtained.

### 3.2 Comparison of Flavonoid Extraction Methods

The residue of OP from EO extraction (OP-HD) was used to extract flavonoids with several methods including conventional SE, UE, and sequential USE extraction. Table 2 shows the TPC, TFC, and antioxidant activities of the flavonoids obtained from different extraction techniques, where the extraction method had a significant impact. It was obvious that flavonoids extracted by the UE approach had significantly higher TFC and antioxidant capacity (measured by the DPPH assay) than SE and USE methods (*p* < 0.05), as a result of extended UAE durations, which encourage more breakdown of the solid vacuole and cell wall, allowing solvent penetration and biocompound diffusion.^76^ USE yielded significantly lower TPC, TFC, and antioxidant activity when compared to the other two methods. This is believed to be a result of increased temperature due to ultrasound treatment, which was reported to be as high as 48 °C when cooling is not implemented.^77^ In a study conducted by Zimare et al.,^32^ phenolics from *Lobelia nicotianifolia* leaves were extracted with the assistance of the UAE; however, at higher temperatures and longer extraction times, a decline in TPC and TFC was observed.^32^ Improved tissue disruption overcoming the negative effects of temperature increase is thought to be the reason for UE performing significantly better than USE. Hesperidin, naringin, narirutin, and neohesperidin are the most prevalent citrus flavonoids.^78^ Hesperidin has a wide range of biological properties, including anti-inflammatory, antibacterial, antioxidant, and angiogenic activity. Additionally, hesperidin has been extensively used to treat inflammation, allergies, liver diseases, and acceleration of wound healing. From this point of view, the hesperidin content is taken into account for the selection of the extraction method. Similar results have been reported by several researchers.^37,79−83^ The positive effect of ultrasound on bioactive compounds was linked to improved penetration of solvent into the plant matrix, plus improved diffusion of the compound of interest into the solvent, both of which are due to jet impacts of cavitation bubbles when collapsed, as the main driving force for UE, as well as due to possible pore enlargement of the plant tissue.^84^

**Table 2:** TPC, TFC, Antioxidant Activity by DPPH, and CUPRAC Assays of Flavonoids Obtained by Different Extraction Methods^a^

extraction method	TPC (mg GAE/g OP-HD)	TFC (mg RE/g OP-HD)	antioxidant activity by DPPH assay (mg TEAC/g OP-HD)	antioxidant activity by CUPRAC assay (mg TEAC/g OP-HD)
SE	4.77 ± 0.65^a^	1.87 ± 0.24^b^	3.16 ± 0.09^b^	7.31 ± 0.59^a^
UE	4.67 ± 0.18^a^	2.50 ± 0.01^a^	3.70 ± 0.07^a^	6.93 ± 1.14^a^
USE	1.29 ± 0.17^b^	1.07 ± 0.34^c^	2.07 ± 0.19^c^	5.68 ± 0.18^a^

a GAE: gallic acid equivalent; RE: rutin equivalent antioxidant capacity; and TEAC: Trolox equivalent antioxidant capacity. Means with different letters at each column are significantly different (*p* < 0.05).

To summarize, because of cavitation and the quick creation and collapse of air bubbles that rupture the cell wall and release the phenolic compounds, ultrasonication-based extraction yielded a greater recovery of the phenolic compounds.

Hesperidin and neohesperidin were the most prevalent flavonoids in the obtained extracts (Table 3). Rutin contents were significantly improved by UE and USE methods (*p* < 0.05). Flavonoids were higher in OP extracts from UE than SE. Wang et al.^37^ also reported that flavonoid concentrations and phenolic fractions were generally higher in UE and enzyme-assisted extraction than conventional SE from brocade orange (*C. sinensis*) peels. There were no significant differences between UE and USE on neohesperidin content, while hesperidin content was significantly higher by UE compared to other methods, similar to previous results,^37,85,86^ probably due to improved extraction as discussed above. The reason why higher concentrations of flavonoids are obtained as a result of UE treatments is that the interactions such as covalent and hydrogen bonds that keep the phenolic components bound to the food matrix are destructed.^83^

**Table 3:** Flavonoid Concentrations Obtained by Different Extraction Methods^a^

extraction methods	mg/g citrus peel		
	rutin	hesperidin	neohesperidin
SE	0.31 ± 0.08^c^	1.41 ± 0.18^b^	2.37 ± 0.14^b^
UAE	0.74 ± 0.09^b^	3.58 ± 0.08^a^	4.07 ± 0.46^a^
USE	1.26 ± 0.28^a^	0.83 ± 0.04^c^	3.49 ± 0.34^a^

a Means with different letters at each column are significantly different (*p* < 0.05).

After the selection of the appropriate method as UE, optimal extraction conditions were investigated based on TPC, TFC, and antioxidant activity, as well as individual concentrations of rutin, hesperidin, and neohesperidin (Table 4). Previous studies showed that flavonoid extraction yield varied by the fruit type. About 8.23–15.56 g/100 g, 3.26–8.93 g/100 g, 8.66–13.56 g/100 g, and 7.43–12.73 g/100 g were extracted from sweet orange, lemon, tangerine, and grapefruit peels, respectively.^7^ Additionally, Selahvarzi et al.^87^ reported that the extraction yield of OP was 11%, and our extraction yield was 20.89%, higher than the study. In another study conducted by Chen et al.,^64^ five different OP source extraction yield ranged from 27.3 to 41.4% (w/w) of dried OP, with hot alkaline water extraction. Flavanone yield from orange (*C. sinensis* L.) peel by ethanol extraction was 6.27–10.03%.^80^ Dalmau et al.^89^ observed that orange byproduct extraction yields were 22, 19, and 13% at 5, 15, and 25 °C with ultrasound extraction, respectively. These differences should be explained with the differentiation of extraction methods and solvents.

**Table 4:** Factorial Design with the Observed TPC, TFC, Antioxidant Capacity, and Individual Flavonoid Concentrations^a^

time (min)	solvent-to-solid ratio (v/w)	TPC (mg GAE/g OP-HD)	TFC (mg rutin/g OP-HD)	antioxidant activity by DPPH assay (mg Trolox/g OP-HD)	antioxidant activity by CUPRAC assay (mg Trolox/g OP-HD)	rutin (mg/g OP-HD)	hesperidin (mg/g OP-HD)	neohesperidin (mg/g OP-HD)
50	40	2.84 ± 0.53^b^	8.14 ± 3.03^b^	2.03 ± 0.64^c^	5.87 ± 0.73^c^	0.69 ± 0.76	2.51 ± 2.21	3.15 ± 2.25
	80	6.29 ± 1.06^a^	11.7 ± 1.71^ab^	5.76 ± 1.39^b^	9.8 ± 2.24^b^	6.02 ± 3.73	10.81 ± 6.45	5.20 ± 2.16
	120	7.32 ± 0.65^a^	16.2 ± 1.04^a^	8.23 ± 2.46^a^	13.41 ± 3.53^a^	5.13 ± 1.08	14.41 ± 3.53	5.36 ± 2.7
60	40	2.97 ± 0.36^b^	10.01 ± 0.98^b^	2.59 ± 1.15^c^	4.92 ± 0.58^c^	3.16 ± 1.66	8.49 ± 2.97	2.65 ± 0.11
	80	5.32 ± 0.32^a^	11.03 ± 1.02^ab^	4.43 ± 1.48^b^	8.35 ± 1.62^b^	6.92 ± 1.42	14.02 ± 2.73	4.38 ± 0.55
	120	6.4 ± 0.43^a^	14.78 ± 2.36^a^	6.94 ± 2.09^a^	10.96 ± 1.74^a^	5.67 ± 0.34	3.31 ± 1.73	14.88 ± 2.5
70	40	3.57 ± 0.45^b^	6.67 ± 3.42^b^	2.13 ± 0.34^c^	6.74 ± 1.44^c^	2.37 ± 1.87	3.05 ± 1.9	3.76 ± 1.71
	80	6.28 ± 2.74^a^	10.29 ± 0.65^ab^	4.91 ± 1.61^b^	9.42 ± 1.71^b^	0.47 ± 0.32	3.02 ± 1.27	3.22 ± 1.06
	120	7.15 ± 0.91^a^	13.04 ± 3.06^a^	6.31 ± 1.61^a^	9.27 ± 2.31^a^	2.94 ± 1.8	9.35 ± 3.38	5.56 ± 1.48

a Means with different letters at each column are significantly different (*p* < 0.05).

Statistical analysis revealed that solvent-to-solid ratio had a significant effect on all dependent variables (*p* < 0.05). When solvent proportion was increased, TPC, TFC, and antioxidant capacity of flavonoids increased as well; several researchers reported similar patterns in their study.^37,87,90^ The reason behind this effect was thought to be similar to our previous discussion regarding the effect of water amount for EO extraction, which was the improved diffusion of compounds into the solvent due to a larger concentration gradient at higher solvent-to-solid ratios.

When individual concentrations of selected flavonoids were examined (Table 4), both of the independent variables as well as their interactions were statistically significant (*p* < 0.05). Extending the treatment period caused a decrease in hesperidin and rutin concentrations. Prolonged exposure to ultrasonication results in structural damage to the solute and degradation of bioactive chemicals. It was reported that at higher temperatures and/or for longer periods of time, the contents of hesperidin decreased to varying degrees.^91^ Hesperidin contents were reported in the range of 8.19–20.17 mg/g dw in *C. sinensis* pulp.^90^ Hesperetin is converted into the tasteless hesperidin and its bitter isomer neohesperidin by the actions of 1,6-Rhat or 1,2-Rhat, respectively, both of which are widely distributed in OP, despite the fact that typically only one isomer exists at a predominate level. For instance, *Citrus grandis* peels from Wendun contained a high amount of neohesperidin,^92^ while in sweet oranges (*C. sinensis*)^64^ and brocade orange (*C. sinensis* L. Osbeck), *Citrus reticulata*(88) was the most abundant.^86^ Similar patterns are also shown in Table 4.

Considering data in both Tables 3 and 4, the selected conditions for the flavonoid extraction were determined as the solvent-to-solid ratio of 120 mL/g and an extraction time of 50 min. Rutin, hesperidin, and neohesperidin contents of flavonoids at selected conditions were 2.31 ± 0.58, 14.25 ± 3.39, and 4.02 ± 0.87 mg/g OP-HD, respectively.

It is also important to discuss the feasibility of extraction methods in addition to knowing that the added value of bioactive compounds is high.^93,94^ The current study showed that the ultrasound-assisted approach resulted in a bioactive component with higher bioactivity. The equipment cost of the UAE method is lower compared to suggested novel methods in the literature, and it also uses less energy during short-term extraction. In addition, the automated system has the benefit of requiring fewer workers than conventional techniques.^95^ Apart from its economic aspect, the UAE method is notable due to its support for sustainable food system and being environmentally friendly.^96^ In spite of the fact that disposing of one ton of solid waste in Europe costs around between 28 and 60 US $,^97^ since the payback period of the UAE system is less than a year, it is understood that UAE method offers a significant economic advantage in addition to the higher biological activities of bioactive compounds.^98^ UAE, which is stated to be applied in orange juice byproducts, has the potential to have a significant place in the evaluation of the these wastes, which is around 15 million tons per year, and the related sustainability issue.^99^

### 3.3 Bioactivity of EO and Flavonoids under the Selected Extraction Conditions

Total phenolic and flavonoid contents, antioxidant capacity with DPPH and ABTS assays, and antimicrobial activity of EO and flavonoids extracted under selected conditions were evaluated (Table 5). In accordance with our results, Değirmenci and Erkurt^100^ determined TPC of EO as 1.54 ± 0.08 mg GAE/g EO. Raspo et al.^101^ investigated the orange EO antioxidant activity with several methods, including DPPH, CUPRAC, FRAP, and ABTS assay. Nearly, 8 mg of Trolox/mL of EO and 2.5–4.3 mg of Trolox/mL of EO capacity with DPPH and CUPRAC assay were reported, similar to our results. DPPH radicals can be deactivated through two methods: by either hydrogen atom-transfer or single-electron-transfer (SET) mechanisms. The antioxidant activity of the CUPRAC test relies on the reduction of copper(II) to copper(I) facilitated by the SET mechanism.^102^ These results show that the antioxidant activities of flavonoids were consistently higher than those detected by the DPPH assay (Table 4). It means that EO and flavonoids in this study exhibit higher antioxidant qualities in reduction capacity compared to free-radical scavenging activity.

**Table 5:** Bioactivity of EO and Flavonoids Obtained under Selected Extraction Conditions^a^

	TPC	TFC	antioxidant activity by DPPH assay	antioxidant activity by CUPRAC assay
EO	3.07 ± 0.19 (mg GAE/mL EO)	29.48 ± 2.48 (mg RE/mL EO)	3.82 ± 0.09 (mg TEAC/mL EO)	5.50 ± 1.37 (mg TEAC/mL EO)
flavonoids	8.35 ± 1.55 (mg GAE/g OP-HD)	6.48 ± 3.03 (mg RE/g OP-HD)	10.61 ± 0.32 (mg TEAC/g OP-HD)	10.61 ± 3.15 (mg TEAC/g OP-HD)

a Extraction conditions for EO: 5.5 mL water/g OP and HD time of 180 min. Extraction conditions for flavonoids: 50 min of UE with 120 mL solvent/g OP-HD.

Additionally, Manzur et al.^103^ determined antioxidant capacity and TPC of orange EO as 10.53 ± 1.20 μg GAE/mL EO and 0.52 ± 0.006 mg TEAC/mL, respectively, which were lower than our results. The antioxidant activities of various species of citrus peel EOs vary. The percentage variation of the main chemical compounds found in the extracted plant EO was most likely the cause of this difference.^63^ The results in different cultivars of oranges were also different, such as bitter orange (IC_50_ = 19.6–200 μg/mL),^104^ navel orange (IC_50_ = 9.45 μg/mL),^105^ sweet peel orange (375–643 mg Trolox/g EO),^10^ and bitter OP (5.23 mg GAE/mL EO).^106^ Numerous antioxidant assays were used due to antioxidants’ complexity and reactivity. It is not surprising that the relative activities with these antioxidant tests will vary, given the complexity of these mixtures and the various test principles.

MIC and MBC values of orange EO and flavonoids obtained under selected conditions against five different microorganisms are shown in Table 6. While the MIC and MBC values of EO were higher than those of flavonoids, the lowest-resistant microorganism was noted as *S. pyogenes* ATCC 19615 against both bioactive fractions. In general, it was reported that the effectiveness of EO against Gram-positive microorganisms should be greater than Gram-negative microorganisms due to their difference in the cell walls’ structure which explains the sensitivity of *S. pyogenes* ATCC 19615.^101^ However, on the contrary, EO (MIC: 0.39–3.13 μL/mL) was found to be more effective against Gram-negative microorganisms.^70^

**Table 6:** MIC and MBC Values of EO and Flavonoids Obtained under Selected Extraction Conditions^a^

microorganism	EO		flavonoids	
	MIC (mg/mL)	MBC (mg/mL)	MIC (mg/mL)	MBC (mg/mL)
E. coli ATCC 25922	256	256	64	64
P. aeruginosa ATCC 27853	256	256	64	64
S. aureus ATCC 6538	128	256	16	16
S. aureus ATCC 43300	256	256	16	16
S. pyogenes ATCC 19615	32	32	8	8

a Given in Table 5.

In other studies, while the MIC value of sweet orange EO was determined between 4.66 and 18.75 μL/mL,^107^ Torres-Alvarez et al.^108^ determined MIC and MBC values in the range of 0.5–2 mg/mL and 2 → 2.5 mg/mL, respectively. These variations in antibacterial activity might be due to the method used for extraction, the type of orange, or the part of the orange used. For example, Geraci et al.^109^ exposed *Listeria monocytogenes* to EO obtained from the Sanguinello Paternò (SPP) and Moro Solarino (MSP) orange peels. They concluded that the inhibitory effect of the MSP source EO (92 mg/mL) was approximately 6 times lower than that of SPP (15 mg/mL).^109^ Besides, MIC values against two strains of *S. aureus* and *P. aeruginosa* were indicated as higher than 100 mg/mL in accordance with the current study.

Flavonoids, which have been shown by various studies to be in the composition of OP,^88,106^ have various biological roles, one of which is their antimicrobial effect.^110^ It is known that they present this effect with different mechanisms,^111,112^ and rutin and hesperidin-neohesperidin, which are included in the extracted flavonoid content, have been shown to have effects such as membrane-disrupting and inhibition of biofilm formation, respectively.^113^ While studies generally focus on the antimicrobial effect of a specific component in the flavonoid composition, MIC values against different bacteria were shown as >1000 μg/mL for neohesperidin, ≤1000 μg/mL for hesperetin,^114^ and 500–1000 μg/mL for rutin.^115^ The inhibitory concentration may be higher as it is known that a wide variety of components are included in the extracted flavonoid composition.

Besides, antimicrobial differences result from variations in the antimicrobial components present in the resultant bioactive agent’s composition. The amount of d-limonene in orange EO, which is known to have antibacterial properties,^116^ varies depending on the orange variety,^109^ and this circumstance has a significant impact on the effectiveness against pathogens as seen in flavonoid.

### 3.4 Extraction and Characterization of Pectin

Pectin is a valuable component with a wide range of uses including food, cosmetic, and medicine.^28^ Therefore, pectin that was solubilized in hot water during HD was extracted with a yield of 3.19 ± 0.03 g/g OP. On the other hand, the yield of pectin directly extracted from orange juice waste was around 13–14 g/100 g dry matter.^49^

While the DE of extracted pectin was found as 54.25% analogous with the study of Saberian et al.,^49^ the quality of pectin was evaluated as high with a DE greater than 50%. On the other hand, Fidalgo et al.’s^28^ work resulted in the DE of orange pectin as 35–36% which is lower than the current study. Since the DE impacts the gelling ability of pectin, high methoxyl pectin (DE > 50%) is preferred in the food industry.^23^ Thus, extracted pectin has the potential to be highly evaluated in various prospective studies. Besides the DE, pectin should include a certain amount of GalA, and the GalA content present in the pectin was 22.06 ± 1.72%. For the polymer to be considered commercial pectin, it must contain at least 65% GalA.^23^ Although the result obtained is different than expected, it is stated that the GalA content is also affected by various extraction conditions.^48^ Also, while the DE of OP was between 1.7 and 37.5%, which is lower than the current study, the GalA content was 71.0% under optimum conditions, with a DE of 1.5%. From these results, it can be understood that DE was not related with the extraction yield.^51^

**Figure 2:**
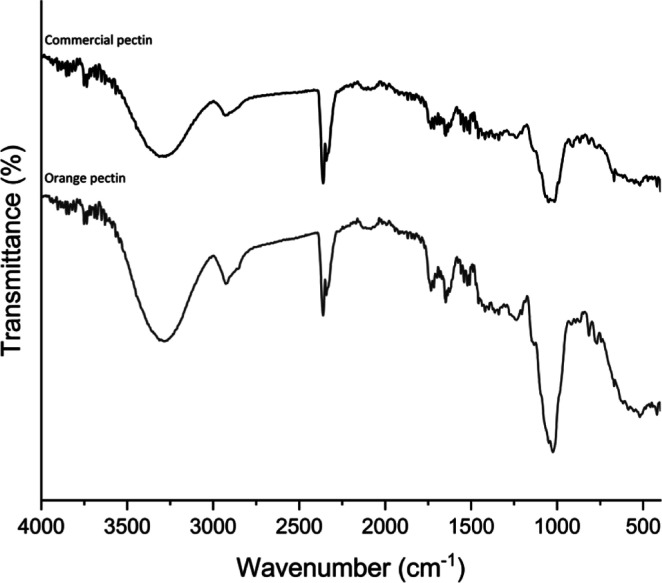
FT-IR spectra of orange and commercial pectin.

In Figure 2, the FT-IR spectra of produced orange pectin and commercial pectin are shown. FTIR analysis was used to assess the structural characteristics of produced pectin. Pectin can be classified into high-methoxylated pectins (HMPs, DM > 50%) and low-methoxylated pectins (LMPs, DM < 50%). The wavelength range of 950–1200 cm– 1, referred to as the carbohydrate fingerprint zone, offers important information about the existence of key functional groups in polysaccharides.^117^ The results show that this study effectively extract pectins from OP powder, when compared with commercial pectin FTIR, due to similar spectra. The only difference is the intensity of the peaks, similar to Du et al.^38^ studies. The characteristic peaks of pectin at 1748 and 1630 cm^–1^ can also be used to distinguish whether the pectin is HMP or LMP. The pectin obtained in this experiment was weakly absorbed at 1748 cm^–1^ and strongly absorbed at 1630 cm^–1^, so the produced pectin was specified as LMP, which was also proved by the methoxylated determination of pectin by titration, similar to the study conducted by Qi et al.^118^ that investigated the citrus pectin. The GalA methyl esters’ CH, CH_2_, and CH_3_ lengths are responsible for the absorbance at about 2900 cm^–1^. The peak at 1740 cm^–1^ is the C=O stretch observed in the ester and derived from the acetyl (COCH_3_) group. The bands at 1380–1445 cm^–1^ denote the existence of CH_3_ groups, while the peak at 1630 cm^–1^ is associated with the OH tensile vibration band. The bands at 1015–1100 cm^–1^ belong to C–O bending or stretching.^119^ It has been observed that while the produced OP pectin has characteristic 1630 and 1748 cm^–1^ peaks, it also includes GalA methyl ester bonds. The peaks between 1200 and 900 cm^–1^ represent prominent and distinctive bands for glucose, fructose, and sucrose^120^ which are also seen in the OP pectin. Eventually, it was concluded that the peaks were found compatible with the range in the literature to compare with other produced citrus pectin.^24,49,118^

The data confirm that orange pectin is comparable to commercial pectin. Pectin is particularly ideal for usage in the gelly and candy sector due to its high degree of methylation.

Moreover, high methoxy pectin is primarily used to stabilize specific types of sour milk products. From the point of view, produced pectin may be used on confectionery and dairy-based food products and various citrus peel pectins have been studied for their role as components in food-packaging films.^117,121^

## 4 Conclusions

The effects of extraction conditions for EO (water-to-solid ratio and HD time) as well as for flavonoids (solvent-to-solid ratio and extraction time) were thoroughly investigated to maximize yield and d-limonene concentration for the former and higher bioactivity of selected flavonoids for the latter. Ultrasonication improved both bioactivity and flavonoid concentrations when applied in the extraction of flavonoids on the remaining solids after EO extraction (OP-HD). UAE is recognized as a quick, cost-effective, environmentally friendly, and efficient way to extract flavonoid fractions from orange juice waste. Both the EO and flavonoid fractions had moderate to high bioactivity. Finally, pectin that was solubilized in hot water during HD was extracted by ethanol precipitation and characterized. Therefore, this study suggests that orange juice waste is a promising candidate for achieving the recovery of value-added d-limonene, natural antioxidants, and pectin.

Overall, this work reports an environmentally friendly approach for valorization of industrial orange waste to produce several components that would be of interest for food, pharmaceutical, and cosmetic industries. Considering the extraction process of orange juice waste from a different angle, it will be interesting to look into factors such as temperature, frequency of sonication, and solvent mixture that are important on an industrial scale and need additional study and development. Future studies might focus on investigating the application of these bioactive compounds to produce biomaterials for the drug, food, and cosmetic industry. Moreover, the safety of the compounds based on orange waste should be investigated. Through the reduction and utilization of fruit waste byproducts that are now being placed in landfills, the sequential extraction of these compounds could help in the transformation of the agricultural industry from a linear economy to a circular economy.

## Supporting Information Available

HPLC chromatogram of d-limonene at 200 nm (PDF)
