# Supplementary material for: Sequential Extraction and Characterization of Essential Oil, Flavonoids, and Pectin from Industrial Orange Waste
Source: ACS Omega. 2024 Mar 16;9(12):14442–54. doi: 10.1021/acsomega.4c00112 (PMC10976415; doi:10.1021/acsomega.4c00112)
Supplement: Supplementary file 1 — ao4c00112_si_001.pdf [file ao4c00112_si_001.pdf]

Sequential extraction and characterization of essential oil, flavonoids and pectin from industrial orange waste

Dilara Nur Dikmetas[1], Dilara Devecioglu[1], Funda Karbancioglu-Guler[1], Derya Kahveci[1]*

Author affiliation

[1]Istanbul Technical University, Faculty of Chemical and Metallurgical Engineering, Department of Food Engineering, Maslak, 34469 Istanbul, Türkiye

Contact information for Corresponding author

Istanbul Technical University, Faculty of Chemical and Metallurgical Engineering, Department of Food Engineering, Maslak, 34469 Istanbul, Türkiye. kahvecid@itu.edu.tr

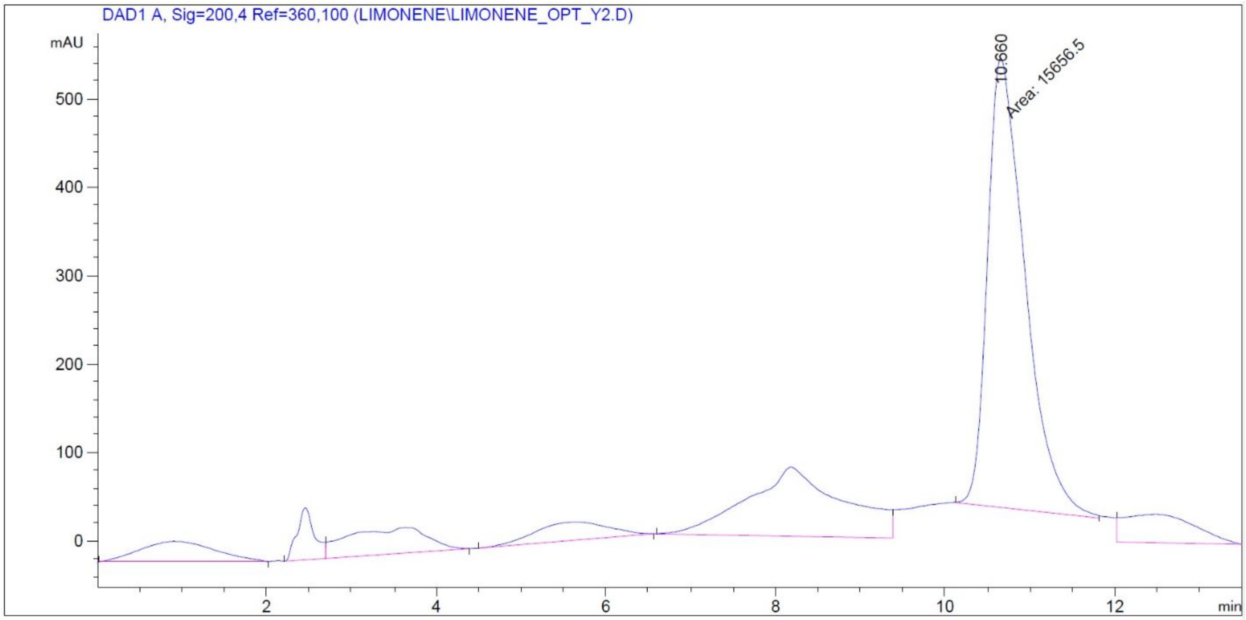

Figure S1. HPLC chromatogram of the D-limonene at 200 nm
